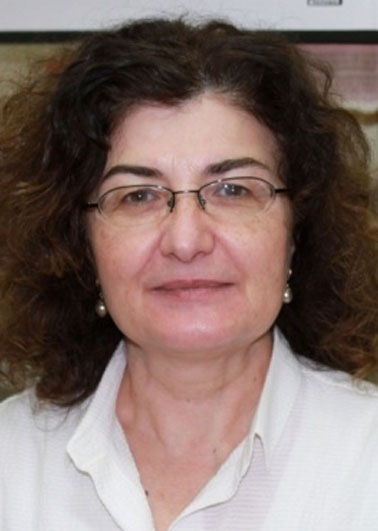# Photochemistry in the Low‐Temperature Processing of Metal Oxide Thin Films by Solution Methods

**DOI:** 10.1002/chem.202000244

**Published:** 2020-06-08

**Authors:** Iñigo Bretos, Ricardo Jiménez, Jesús Ricote, M. Lourdes Calzada

**Affiliations:** ^1^ Instituto de Ciencia de Materiales de Madrid (ICMM-CSIC) C/ Sor Juana Inés de la Cruz 3 Madrid 28049 Spain

**Keywords:** low-temperature processing, photocatalysis, photochemical solution deposition, photolysis, thin films

## Abstract

Photochemistry has emerged in the last few years as a powerful tool for the low‐temperature processing of metal oxide thin films prepared by solution methods. Today, its implementation into the fabrication procedure makes possible the integration of amorphous semiconductors or functional crystalline oxides into flexible electronic systems at temperatures below 350 °C. In this review, the effects of UV irradiation at the different stages of the chemical solution deposition of metal oxide thin films are presented. These stages include from the synthesis of the precursor solution to the formation of the amorphous metal‐oxygen network in the film and its subsequent crystallization into the oxide phase. Photochemical reactions that can be induced in both the solution deposited layer and the irradiation atmosphere are first described, highlighting the role of the potential reactive chemical species formed in the system under irradiation, such as free radicals or oxidizing compounds. Then, the photochemical effects of continuous UV light on the film are shown, focusing on the decomposition of the metal precursors, the condensation and densification of the metal‐oxygen network, and the nucleation and growth of the crystalline oxide. All these processes are demonstrated to advance the formation and crystallization of the metal oxide thin film to an earlier stage, which is ultimately translated into a lower temperature range of fabrication. The reduced energy consumption of the process upon decreasing the processing temperature, and the prospect of using light instead of heat in the synthesis of inorganic materials, make photochemistry as a promising technique for a sustainable future ever more needed in our life.

## Graphical abstract

**Photochemistry** has emerged as a powerful tool for the low‐temperature processing of metal oxide thin films prepared by solution methods leading to 1) the synthesis of low‐temperature liquid precursors, 2) an enhanced decomposition of metal precursors, 3) a high degree of condensation and densification of the metal‐oxygen network, and 4) a prompt nucleation and growth of the crystalline phase.

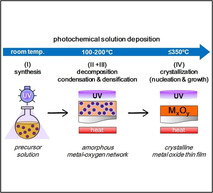

## 1 Introduction

Photochemistry is defined as the branch of chemistry concerned with the chemical effects of light (ultraviolet, visible, or infrared radiation). Since light is actually a form of radiant energy, a compound can be promoted from its ground state to an excited state of energy upon light absorption. This may produce several chemical (also physical) processes according to the Grotthuss–Draper law, that is, the first law of photochemistry. Thus, the excited‐state energy can induce photochemical reactions such as elimination, cleavage, rearrangement, isomerization, cyclization, addition, or electron transfer.^1^ Such reactions constitute the basis of organic photochemistry, which contributed to accelerate the synthetic chemistry methodology as it is known today.^2^ Organic photochemistry enables unique pathways for the synthesis of compounds not thermodynamically favored nor easily accessible by other methods.^3^

However, during the last few years photochemistry has gained attention in the field of inorganic chemistry, particularly in the low‐temperature processing of metal oxide thin films.^4^, ^5^, ^6^, ^7^ To understand how this discipline has evolved up to reaching the former point, we must first consider the eruption of flexible electronics at the beginning of this century (2000s).^8^ This emerging technology, often referred to as the next ubiquitous platform of our lives, involves the fabrication of large‐area electronic devices (e.g., visual displays, solar cells, smart textiles, or electronic skin) integrated on lightweight and low‐cost flexible systems based on plastic, rubber, or even paper.^9^ Polymer materials such as polyimide (PI), polyethylene terephthalate (PET), polyethylene naphthalate (PEN), and polycarbonate (PC) are the most widely used substrates by this technology. However, their relatively low thermal stability (thermal degradation or glass transition several hundreds of degrees Celsius below for example, rigid glass or silicon wafers) drastically limits the temperature at which the functional layer deposited onto them can be further annealed—usually not beyond 350 °C. Due to their low processing temperatures and “soft” nature, organic materials (carbon based polymers or organic molecules) were soon considered the ideal candidate materials for their growth on this type of substrates giving rise to the so‐called organic electronics. In spite of the higher performance and stability of their inorganic (mostly oxide based) counterparts, the relatively high processing temperatures of most metal oxide layers prevented their direct integration into flexible electronic systems at first instance.

**Figure 1:**
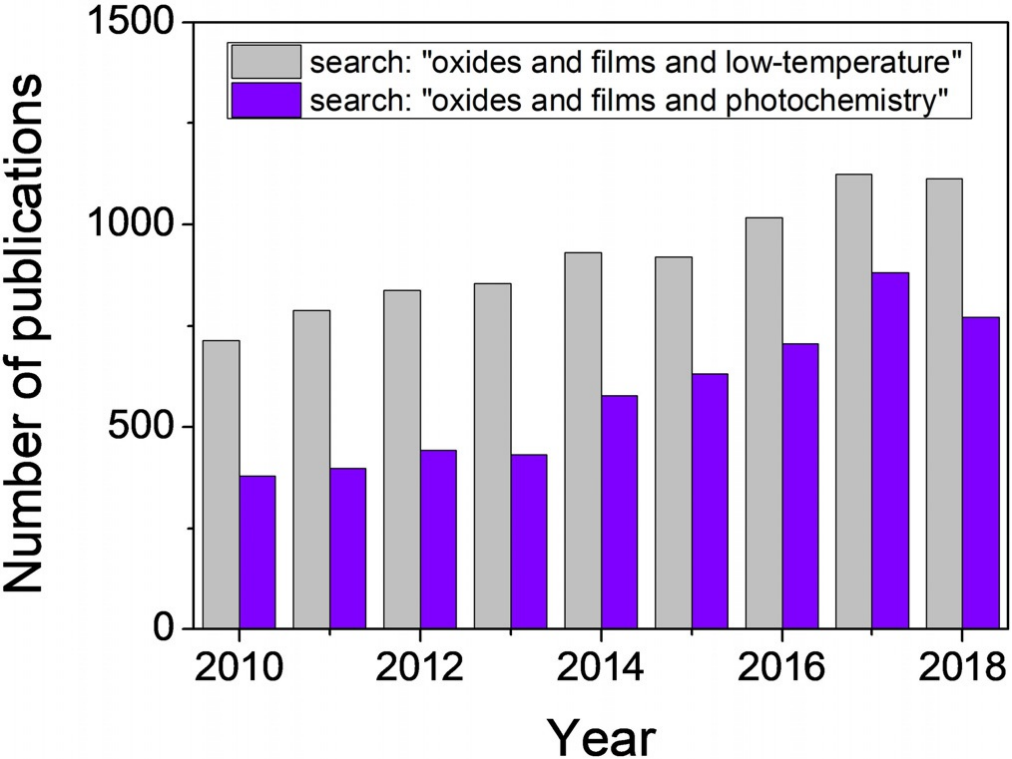
Number of publications on low‐temperature processing of metal oxide thin films reported from 2010 to 2018, highlighting those where photochemistry is addressed to induce either the formation or the crystallization of the metal oxide. Source: Web of Science.

In 2004, high‐performance thin film transistors (TFTs) were demonstrated on amorphous InGaZnO (a‐IGZO) semiconductor oxides.^10^ The study suggested that high field effect mobility and high‐level uniformity could be in principle obtained in metal oxide semiconductors processed at low temperatures despite reaching an amorphous state in the material. This significantly boosted the research on amorphous semiconductors for applications in flexible electronics, since processing temperatures compatible with the polymer substrates could now be applied. Due to their outstanding advantages such as relatively low‐cost, scalability and high deposition rate, solution methods were preferred over vacuum techniques to grow metal oxides on flexible substrates. However, solution‐processed layers require critical processing steps to remove the organic species from the system and to promote the formation of a defect‐free, highly densified metal‐oxygen network. Thermal annealing is in most cases insufficient due to the temperature constraints imposed by the polymer substrate, and complementary strategies to induce the formation of the metal oxide in the film are therefore needed.^6^ Figure 1 shows the number of publications on low‐temperature processing of metal oxide thin films reported in the last ten years, together with those of them where photochemistry is addressed to induce either the formation or the crystallization of the metal oxide. Although the number of papers on this last topic is still moderate, there is an exponentially growing interest on the use of light irradiation to attain metal oxide thin films at low temperatures. The pioneer works on amorphous metal oxide semiconductors (2012) and multifunctional crystalline metal oxides (2014) directly grown on flexible plastic by photochemical methods will probably pave the way for the large number of contributions to this field that are yet to come.^11^, ^12^ Therefore, we foresee an encouraging prospect for this technique in the coming years for the fabrication of next‐generation flexible systems based on new electronic metal oxide materials.^13^ The reduced energy consumption of the process upon decreasing the fabrication temperature, together with the potential sustainability of using light as an alternative energy source instead of thermal heating, make photochemical methods one of the basic alternatives to be considered today for a really green synthesis of materials.^14^, ^15^

But, how can photochemistry aid to the low‐temperature processing of solution‐derived metal oxide thin films? The answer to this question is basically by breaking chemical bonds and facilitating the formation of new ones. Energy is always required to break a bond in a molecule, which is known as bond energy. If chemical bonds are cleaved by light, the decomposition of metal precursors in the system would be favored without the need of thermal annealing. If molecules are cleaved into smaller molecules or individual atoms, new entities such as reactive oxygen species (e.g., radicals) could also be formed upon (photo)chemical reaction. These species can further assist to the elimination of organic residuals from the system, besides promoting condensation and densification of the amorphous metal‐oxygen network. If subatomic particles are generated by light irradiation (e.g,. photogenerated electrons or holes), the acceleration of photoreactions would be induced by photocatalysis, or even the crystallization rate of the amorphous system could be increased.

This review presents the photochemical effects of continuous light irradiation at different stages of the chemical solution deposition process of metal oxide thin films. The most recent advances showing how photochemistry can induce the formation of a defect‐free, highly densified metal‐oxygen network first, and its crystallization into the final metal oxide phase afterwards will be described. Although equally valid in the context of low‐temperature crystallization of metal oxide layers, studies on localized heating by the use of pulsed laser irradiation have not been included due to the absence of a chemical reaction in the system. The primary aim of this review is to provide the fundamental insights of photochemistry that, ultimately, drive the low‐temperature crystallization of solution‐processed metal oxide thin films.

## 2 Photochemical Reactions Induced by Irradiation with UV Light

**Figure 2:**
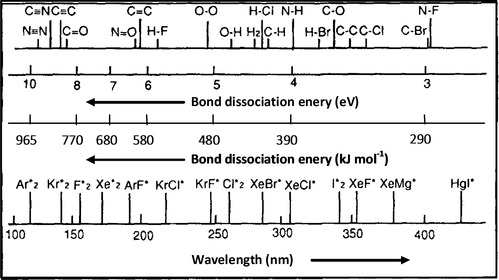
Wavelengths (nm) of commercial excimer lamps with a range of irradiation from the near (354 nm) to the deep (126 nm) UV region. These UV sources are shown in comparison with the energies (eV and kJ mol^−1^) of the typical chemical bonds excited at these wavelengths. Adapted with permission from ref. 20. Copyright 1997 Elsevier Science B.V.

Penetration of UV light is limited to the surface region of condensed matter, typically below 200 nm. Hence, it can be a powerful tool to induce chemical and physical changes in thin film materials.^16^ Irradiation with intense UV light such as that used by pulsed lasers (coherent UV radiation) mainly produces the increase of the local temperature at the film surface, that is, a *thermal excitation*. In contrast, *electronic excitation* is dominant when using continuous irradiation lamps (incoherent UV radiation). Despite these lamps having much lower power than lasers, they can irradiate over large areas under controlled atmospheres thus minimizing thermal excitation and enhancing chemical reactions that are the basis of photochemistry. Figure 2 shows the emission wavelengths of commercial UV sources and the type of chemical bonds that can be excited. Today, we can find commercial excimer lamps with wavelengths in the range of chemical bond energies of many functional groups.^17^ However, photochemical reactions induced by UV irradiation of solution‐deposited layers can take place in two different media: directly in the thin film and in the irradiation atmosphere surrounding the film.

### 2.1 Photochemical reactions in the thin film

Solution (sol‐gel) derived metal oxide layers can absorb the UV light emitted by the aforementioned excimer lamps because they contain molecules that are excited by the energetic photons coming from these sources.^18^, ^19^, ^20^ These photons penetrate into the layer and are consumed by the cleavage of chemical bonds and/or by charge transfer within/between the molecules, and among ions. Therefore, the most common photoreactions that can be produced by UV‐irradiation in the as‐deposited film are *photolysis* and *photoinduced charge transfer*.

Breaking or cleavage of chemical bonds by UV irradiation (generally referred to as photolysis, photodissociation or photodecomposition) easily occurs in solution‐derived layers, since they are actually formed by organic compounds based on metal alkoxides (metal complexes containing organic ligands) and ionic compounds such as organic or inorganic metal salts. Typical bonds present in the former molecular species are summarized in Table 1. It is clear that the energy supplied by excimer UV lamps (Figure 2) is sufficient to produce the dissociation of these chemical bonds. Furthermore, the resulting byproducts can induce subsequent reactions depending on the chemical nature of the photolyzed compounds. Photolysis of organic compounds with long carbon chains results in compounds with shorter carbon chains that would be easily removed from the film by pyrolysis at moderate temperatures. Additionally, during the decomposition of some metal acetates (the most used organic salts in these systems),^21^ the photolysis of intermediate carbonate compounds can result in the formation of free carbonate radicals (CO_3_^.−^)that would assist in the elimination of organic species from the film due to their strong oxidative character [Eq. (1)]:^22^

(1) $$\mathrm{CO}{}_{3}{}^{2-}\overset{hv}{\underset{}{\to }}\mathrm{CO}{}_{3}{}^{•}{}^{-}+e{}^{-}$$

**Table 1:** Types of chemical bonds usually found in the as‐deposited layers derived from precursor solutions of metal oxides.

Chemical Bond	Dissociation Energy		Wavelength
	[kJ mol^−1^]	[eV]	*λ* [nm]
N≡N	941	9.75	127
C=O	805	8.34	149
N=N	631	6.54	190
C=C	607	6.29	197
O=O	498	5.16	240
O−H	464	4.81	258
H−H	436	4.52	274
C−H	413	4.28	290
N−H	393	4.07	304
C−O	358	3.71	334
C−C	347	3.60	345
C−N	305	3.16	392
O−O	204	2.11	586
N−O	201	2.08	595
N−N	160	1.65	748

Metal nitrates are typical inorganic salts used in the synthesis of precursor solutions of metal oxides, either by alkoxide or aqueous routes.^23^ Photolysis of nitrate ions is the mechanism whereby nitrate compounds are decomposed under UV irradiation, which is developed through the photoisomerization of nitrate (NO_3_^−^) to peroxonitrate (ONOO^−^) ions followed by the formation of reactive NO_2_^.^and OH^.^ radicals after the photodecomposition of the protonated form of the peroxonitrate ion (ONOOH) [Eq. (2), (3)]:

(2) $$\mathrm{NO}{}_{3}{}^{-}\overset{hv}{\underset{}{\to }}\mathrm{ONOO}{}^{-}$$

(3) $$\mathrm{ONOOH}\overset{hv}{\underset{}{\to }}\mathrm{NO}{}_{2}{}^{•}+\mathrm{OH}{}^{•}$$

NO_2_^.^ and OH^.^ radicals are known to be highly reactive to hydrogen capture followed by a subsequent M−OH activation for the efficient polycondensation among the metal precursors leading to a metal‐oxygen network in the film with a high degree of condensation and densification.^24^

Besides chemical bond cleavage (photolysis), excited species generated in the film upon light absorption can also transfer their energy to ground state species by different processes. One of them involves the photoinduced charge transfer from activated oxygen species (e.g., oxygen with photogenerated electrons) to metal cations (M^*x*+^), thus inducing the photochemical reduction (*photoreduction*) of the metallic center.^25^, ^26^ The reduced metal atoms can migrate in the amorphous matrix and re‐oxidize with the reactive oxygen species, forming the crystalline oxide (M_2_O_*x*_).Another process is based on the photoinduced charge transfer developed in some metal complexes.^27^ Irradiation of these compounds with UV light may induce intra‐ and inter‐ molecular processes based on the shift of the electronic distribution in the molecule after light absorption. These charge transfer transitions may result in different photoreactions that have been of great usefulness in the synthesis of inorganic materials and also in the low‐temperature processing of crystalline metal oxide thin films.^28^, ^29^, ^30^

### 2.2 Photochemical reactions in the irradiation atmosphere

Photochemical reactions are also produced in the gas atmosphere where the thin film sample is subjected to UV irradiation. Here, the primary process is based on the photolysis of gas molecules into reactive chemical species (mainly, free radicals) usually followed by secondary reactions. These may involve the attack of free radicals to the molecules present in the gas atmosphere forming new radicals and new molecular species. All these chemically reactive compounds can react with the thin film material and contribute to accelerate its crystallization by different mechanisms.

The most conventionally used atmospheres in the UV irradiation of metal oxide thin films are pure oxygen (O_2_), air (O_2_, N_2_ and other minor gases) and inert argon (Ar), where dissociation of oxygen and nitrogen typically occurs under UV light.

Photolysis of oxygen produces free oxygen radicals (O^.^) that can react with molecular oxygen forming ozone (O_3_), whose photodissociation yields oxygen and O^.^ radicals again [Eq. (4), (5), (6)]:

(4) $$O{}_{2}\overset{hv}{\underset{}{\to }}2O{}^{•}$$

(5) $$O{}^{•}+O{}_{2}\to O{}_{3}$$

(6) $$O{}_{3}\overset{hv}{\underset{}{\to }}O{}_{2}+O{}^{•}$$

The strong oxidant character of ozone would enhance the decomposition of organic compounds present in the film by *ozonolysis* (oxidation), whereas the presence of O^.^ radicals would compensate for the charge defects of the crystal lattice, thus improving the oxide stoichiometry. Additionally, when water vapor (H_2_O) is also present in an oxygen atmosphere, the reaction between O^.^ radicals and aqueous vapor can result in the formation of free hydroxyl radicals (OH^.^) [Eq. (7)]:

(7) $$O{}^{•}+H{}_{2}O\to 2\;\mathrm{OH}{}^{•}$$

These highly reactive OH^.^ species would facilitate the hydrolysis and condensation of the metal‐oxygen network, and also contribute to the formation of hydroxyl anions (OH^−^) that can promote the incipient crystallization of metal oxide thin films at low temperatures.^31^

On the other hand, photolysis of nitrogen results in its dissociation into free nitrogen radicals(N^.^)that can react with O^.^ radicals (if oxygen is present in the irradiation atmosphere) beginning new cycles of events [Eq. (8), (9), (10)]:

(8) $$N{}_{2}\overset{hv}{\underset{}{\to }}2\;N$$

(9) $$N{}^{•}+2\;O{}^{•}\to \mathrm{NO}{}_{2}$$

(10) $$\mathrm{NO}{}_{2}\overset{hv}{\underset{}{\to }}\mathrm{NO}+O{}^{•}$$

Summarizing, energetic photons coming from UV lamps can excite many types of molecules present both in the thin film and in the irradiation atmosphere. This electronic excitation can induce molecular dissociations, formation of reactive species or structural changes in the metal‐oxygen network. All these phenomena push the chemical system far from equilibrium, making possible processes that are not achieved by means of conventional thermal treatments.

## 3 Photochemical Solution Deposition of Metal Oxide Thin Films

**Figure 3:**
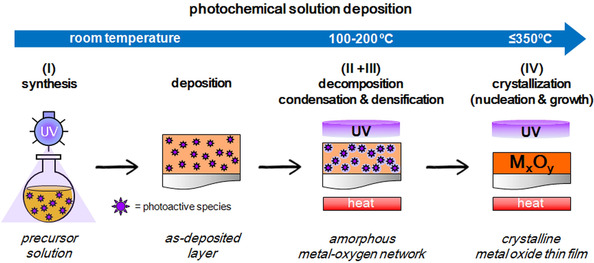
Scheme of the photochemical deposition solution process showing the main steps (I–IV) at which light irradiation can be implemented for the low‐temperature processing of metal oxide thin films.

The first publications reporting the preparation of crystalline metal oxide thin films by chemical solution deposition (CSD) date from the 1980s.^32^, ^33^ Since then, many functional oxide thin films are fabricated by this technique due to particular advantages such as low investment costs, large surface coating, homogeneity and high throughput fabrication.^34^ The method consists first in the synthesis of a stable precursor solution that is deposited on a substrate by a coating technique (e.g., spin‐coating, dip‐coating, spray‐coating). Organic compounds in the resulting layer are then eliminated from the system by thermal treatment (e.g., evaporation, thermolysis, pyrolysis), leading to the decomposition of the respective metal precursors. During this stage, condensation among the metal reagents and densification of the amorphous metal‐oxygen network obtained are gradually developed in the thin film. Crystallization of the functional metal oxide layer is finally carried out by annealing at relatively high temperatures (conventionally between 600–700 °C). However, the versatility of the CSD method has made possible along the years the implementation of complementary techniques in the process, such as the irradiation of the system with UV lamps (see Figure 3). Born in the 2000s, the *photochemical solution deposition* (PCSD) method is originally based on the electronic excitation of the photoactive species present in sol‐gel thin film materials upon the irradiation with light of adequate energy.^16^, ^35^ This can be used for different processes in solution derived films such as patterning, reduction, condensation, densification, or crystallization. However, in the last decade the PCSD method has gained significant relevance in the low‐temperature processing of metal oxide thin films. This is because photochemical reactions induced in the system by light makes possible to attain an appreciable reduction in the processing temperature of the film. In this section, the effects of UV irradiation at each of the different stages (I–IV) of the thin‐film fabrication process by PCSD will be presented. The key processes responsible for promoting both the formation of an amorphous metal‐oxygen network and the further crystallization of the metal oxide thin film down to a lower temperature range will be described.

### 3.1 Photoinduced synthesis of low‐temperature liquid precursors

**Figure 4:**
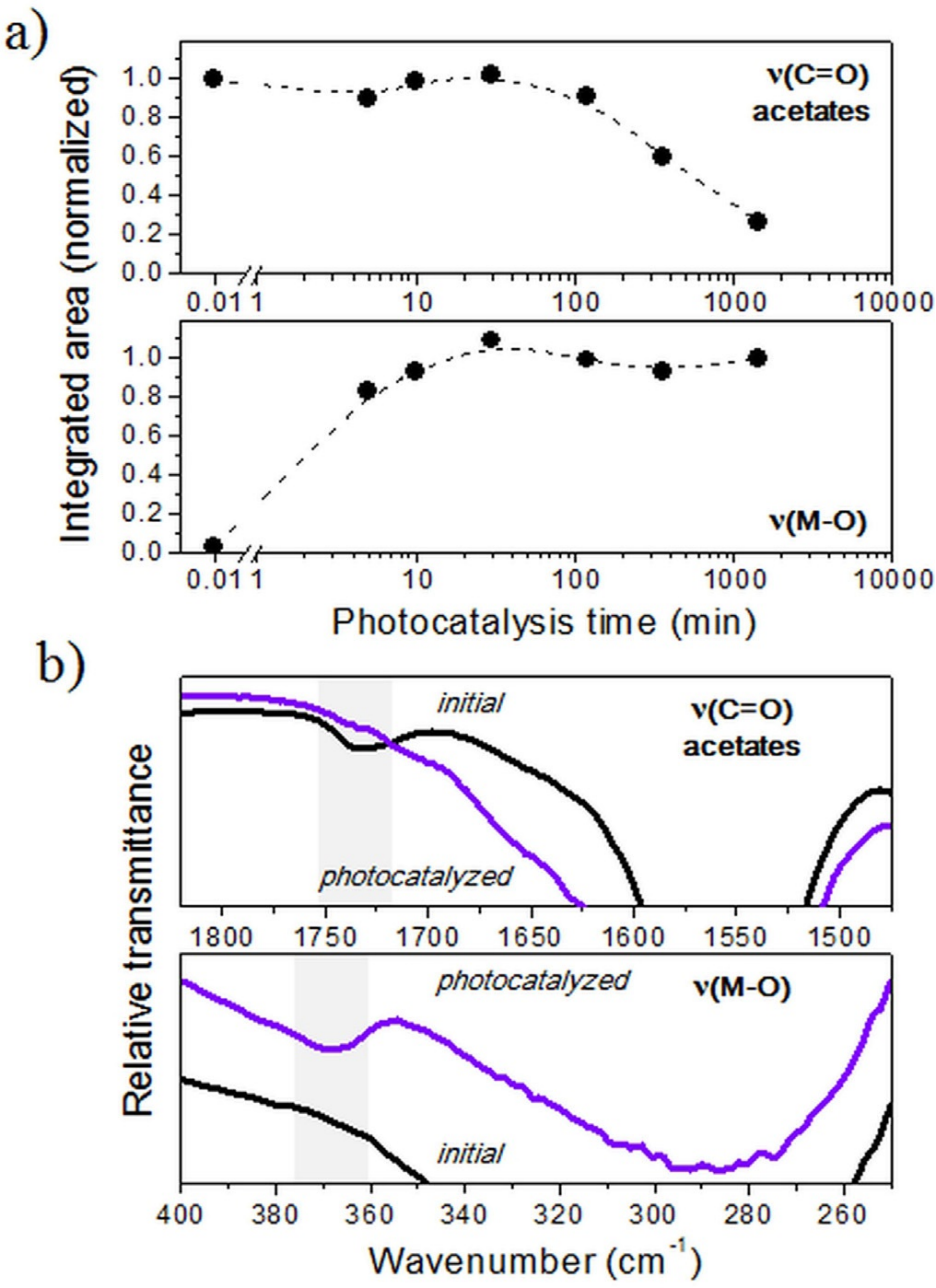
a) Peak area corresponding to FTIR bands associated with ν(C=O) and ν(M−O)modes of a precursor solution of PZT as a function of the photocatalysis time. b) Comparison between the bands at photocatalysis times of 0 min (initial) and 1440 min (photocatalyzed). Adapted with permission from ref. 36. Copyright 2015 Wiley‐VCH.

Most of the works reporting the low‐temperature processing of metal oxide thin films by a photochemical method are actually focused on the effects induced by UV light after the irradiation of the corresponding solution‐derived layers.^4^, ^6^, ^7^ However, few years ago (2015) it was shown that low‐temperature precursors of metal oxides can be directly synthesized in liquid media after the irradiation of the respective precursor solutions containing photocatalytic nanoparticles.^36^ Once these nanoparticles are removed from the solution afterwards, the resulting *low‐temperature liquid precursors* yield metal oxide thin films with a lower crystallization temperature compared to the films derived from the original (initial) solution following the standard steps of a CSD process (deposition, pyrolysis, and crystallization). The proposed concept was developed merging two rather distant fields in materials science; heterogeneous photocatalysis and the low‐temperature processing of metal oxide thin films. The interest in the first scientific discipline accelerated significantly in 1972, when the phenomenon of photocatalytic splitting of water was discovered.^37^ Since then, titanium dioxide (TiO_2_) features predominantly the work on semiconductor photocatalysis with applications addressing many environmental and pollution challenges.^38^ Particularly, hundreds of organic compounds are readily photodegraded today by TiO_2_ photocatalysis (e.g., pollutants, bacteria, tumor cells, etc.).Thus, the novel application of this effect to precursor solutions of metal oxides demonstrated that this photocatalysis assisted method led to the partial decomposition of the organic moieties typically constituent of metal precursors and subsequent polycondensation among metal precursors by an advanced oxidation process carried out at room temperature in liquid media. The investigation was conducted on precursor solutions of ferroelectric Pb(Zr_0.3_Ti_0.7_)O_3_ and multiferroic BiFeO_3_ oxides, to which nanoparticles of TiO_2_ were introduced and the resulting suspensions illuminated with a solar lamp (Osram Ultra‐Vitalux 300 W) to induce the photocatalytic effect. Figure 4 shows the evolution with photocatalysis time of the integrated areas calculated in two representative absorption bands measured by Fourier‐transform infrared spectroscopy (FTIR) at 1734 cm^−1^ [ν(C=O)] and 368 cm^−1^ [ν(M−O)]. The intensity decrease of the first mode is associated with the partial decomposition of organic compounds—in this case, acetates—from the system (Figure 4 a), whereas the substantial rise in the intensity of the second mode accounts for the formation of a metal‐oxygen network that results from the polycondensation among the metal precursors (Figure 4 b).

**Figure 5:**
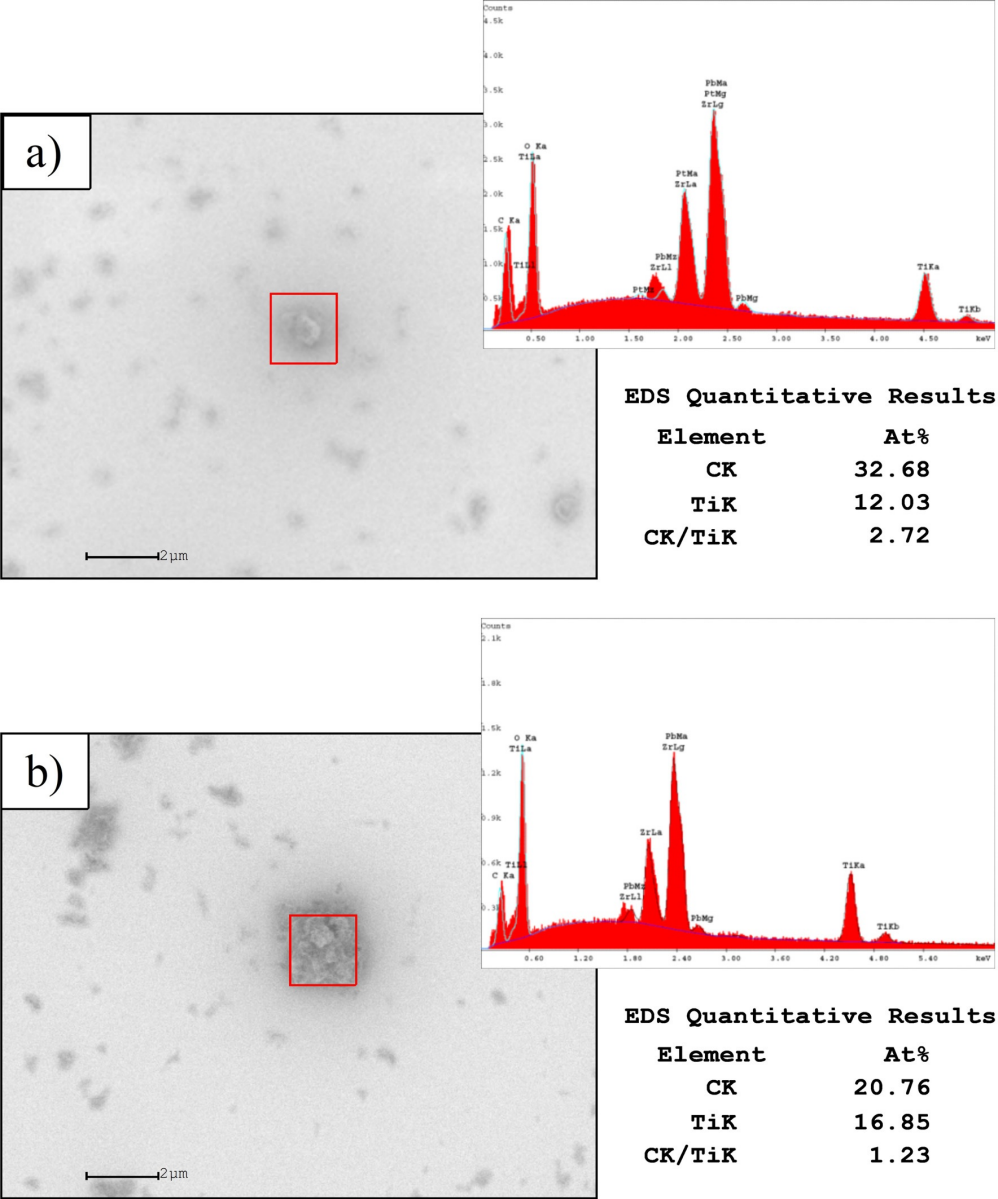
a) Surface image and EDS analysis obtained by SEM on a PZT film with embedded TiO_2_ photocatalytic particles dried at 150 °C and b) subjected afterward to UV irradiation for 1 h at room temperature (Heraeus, Blue Light Excimer System, 222 nm). Reprinted with permission from ref. 36. Copyright 2015 Wiley‐VCH.

**Figure 6:**
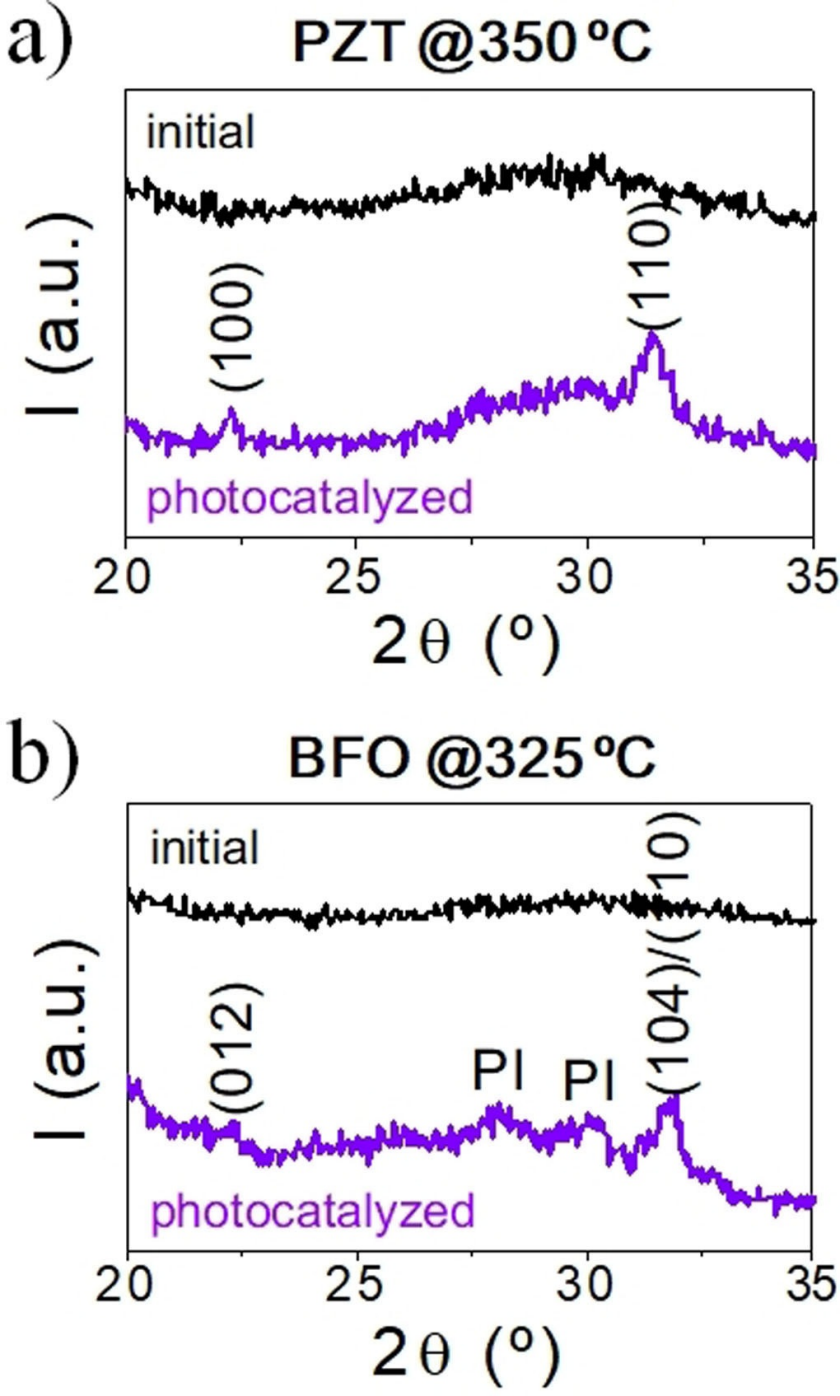
XRD of a) Pb(Zr_0.3_Ti_0.7_)O_3_ (PZT) and b) BiFeO_3_ (BFO) thin films annealed at low temperatures from their respective initial and photocatalyzed precursor solutions. Adapted with permission from ref. 36. Copyright 2015 Wiley‐VCH.

The photocatalytic activity of the TiO_2_ nanoparticles was also investigated in a quasi‐static scenario such as a viscoelastic solid supported on a substrate. Thus, xerogel layers deposited from a suspension of Pb(Zr_0.3_Ti_0.7_)O_3_ containing TiO_2_ nanoparticles were subjected to UV irradiation for 1 h at room temperature. Figure 5 shows the corresponding micrographs obtained by scanning electron microscopy (SEM) in non‐irradiated (Figure 5 a) and irradiated (Figure 5 b) regions of the sample, together with the respective EDS (energy dispersive spectroscopy) analyses. In general, the surface morphology reveals the presence of agglomerated TiO_2_ particles (typically ≈0.1 μm in diameter) within a homogeneous matrix of amorphous matter. To quantify the photo‐oxidation of the residual organic species, the relative amount of carbon surrounding a TiO_2_ agglomerate (expressed as C to Ti ratio relative to the size of the agglomerate) was registered in both irradiated and non‐irradiated regions of the sample. An appreciable decrease of ≈45 % is obtained in the former that accounts for the advanced oxidation of organic species close to the semiconductor surface upon the absorption of UV light. The relatively high degree of decomposition and subsequent polycondensation of metal precursors reached in the solution by this photocatalysis‐assisted process developed at room temperature would be the main feature of this system with respect to the non‐photocatalyzed one. Lower crystallization temperatures are induced in the metal oxide thin films prepared from these low‐temperature liquid precursors (350 and 325 °C for the Pb(Zr_0.3_Ti_0.7_)O_3_ and BiFeO_3_ perovskite systems, respectively) in contrast to the amorphous structures obtained using the same processing conditions for the counterpart films derived from the respective initial solutions (Figure 6).

### 3.2 Photoinduced decomposition of metal precursors

**Figure 7:**
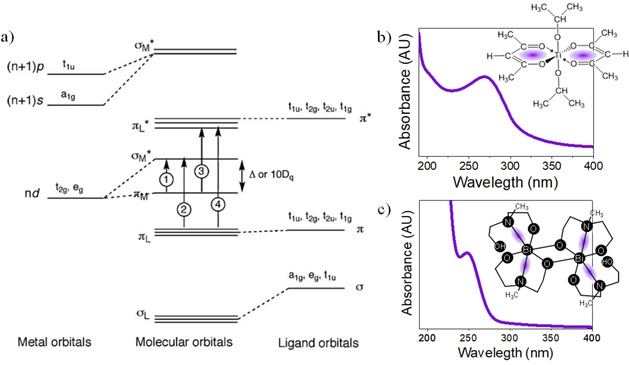
a) Molecular orbital diagram for a transition metal complex showing (1) a metal centered (MC) ligand field transition; (2) a ligand to metal charge transfer (LMCT); (3) a metal to ligand charge transfer (MLCT); and (4) an intra‐ligand or ligand centered (LC) band. Reprinted with permission from ref. 27. Copyright 2011 Elsevier B.V. UV/Vis spectra and molecular structure for photosensitive metal complexes of b) Ti(acac)_2_(OProp^i^)_2_ and c) Bi_2_(mdea)_2_(mdeaH)_2_. Adapted with permission from ref. 35. Copyright 2004 Wiley‐VCH and ref. 29 Copyright 2014 The Royal Society of Chemistry.

Precursor solutions containing photosensitive species may lead to photoactivated thin films once deposited on a substrate and subjected to irradiation with light of adequate energy. To this, metallic centers must react first with organic molecules forming photosensitive coordination complexes that are stable in the solution. Irradiation of these metal complexes in the corresponding gel layers results in intra‐ and inter‐molecular processes upon light absorption involving different electronic transitions such as metal centered (MC), ligand to metal charge transfer (LMCT), metal to ligand charge transfer (MLCT), and intra‐ligand or ligand centered (LC).^27^ Figure 7 shows the molecular orbital diagram for a transition metal complex with the different electronic transitions arisen from light absorption, together with the chemical structure of some photosensitive metal complexes and their ultraviolet‐visible (UV/Vis) spectra measured in solution.^29^, ^35^ The excited states induced in the metal complexes would be responsible for their chemical cleavage into smaller molecular units, according to a chemical process generally known as photolysis. As it was explained in previous Section 2, complementary to the former process the reactive oxygen species generated in the system under UV irradiation (atomic oxygen, ozone, radicals, etc.) can also assist the decomposition of molecular species into small gaseous molecules by rapid radical‐mediated reactions.

**Figure 8:**
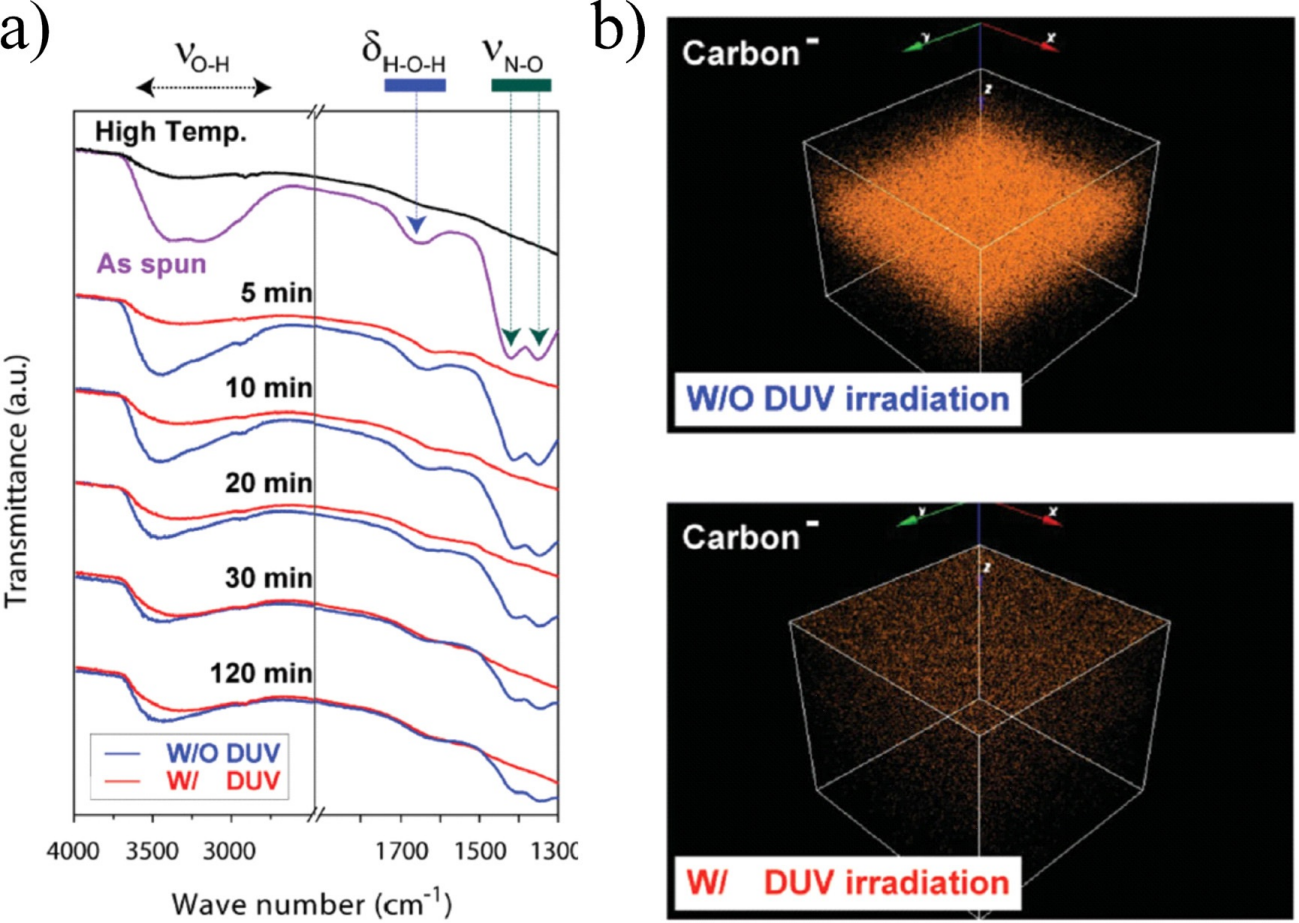
a) FTIR spectra of Al_2_O_3_ thin films maintained at 150 °C without (blue line) and with (red line) DUV irradiation as a function of exposure time. b) TOF‐SIMS 3D mapping images of negative carbon ions inside Al_2_O_3_ thin films maintained at 150 °C without (top) and with (bottom) DUV irradiation. Reprinted with permission from ref. 24. Copyright 2015 Wiley‐VCH.

**Figure 9:**
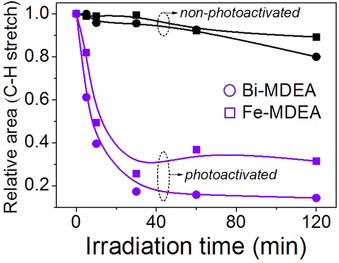
Evolution of the peak area corresponding to the stretching ν(C−H) bands of photoactivated and non‐photoactivated Bi‐MDEA and Fe‐MDEA thin films as a function of irradiation time.

The removal of water molecules and nitrate ligands in thin films derived from a precursor solution of Al_2_O_3_ as a function of the exposure time to UV irradiation at 150 °C can be inferred from the FTIR spectra of Figure 8.^24^ Concerning the broad absorption bands at ≈3500 cm^−1^ [ν(O−H)] and ≈1650 cm^−1^ [*δ*(H‐O‐H)] ascribed to water (Figure 8 a); note how these rapidly decrease in the photoactivated film after irradiation for 5 min reaching similar values to those measured in a film thermally annealed at 350 °C for 1 h. In the case of the absorption bands centered at ≈1400 cm^−1^ and ≈1430 cm^−1^ [ν(N−H)] that correspond to nitrate ligands (Figure 8 a), they practically disappear after the same exposure time to UV irradiation (5 min). In addition, time‐of‐flight secondary ion mass spectrometry (TOF‐SIMS) 3D mapping of residual carbon in these films (Figure 8 b) revealed that the content of this element was dramatically reduced in the entire film thickness upon photoactivation, whereas a substantial amount of carbon was still confined inside the thermally annealed film without photoactivation. Both results reported in this work confirm that deep ultraviolet (DUV) irradiation effectively decomposes the nitrate ligands and solvent molecules present in the as‐deposited films into smaller and diffusible molecules due to direct photodecomposition (photolysis) or complementary radical‐assisted photoreactions. In a more recent study (2020),^39^ a photosensitive metal complex formed between either bismuth or iron with *N*‐methydiethanolamine (MDEA) has been used to induce the crystallization of BiFeO_3_ perovskite thin films at relatively low temperatures (325–350 °C) by UV irradiation. Figure 9 depicts the evolution with irradiation time of the integrated areas corresponding to the C−H vibration bands measured by FTIR spectroscopy in the respective as‐deposited Bi‐MDEA and Fe‐MDEA thin films annealed at 150 °C. The photochemical cleavage of the C−H bond present in organic compounds such as alkanes can be easily deduced from the clear decrease observed in the calculated areas. This result supports the effect of UV light on the decomposition of the organic species present in the system (alkanes). Note that when the film is not photoactivated, the content of organic compounds remains practically constant.

### 3.3 Photoinduced condensation and densification of amorphous metal‐oxygen network

**Figure 10:**
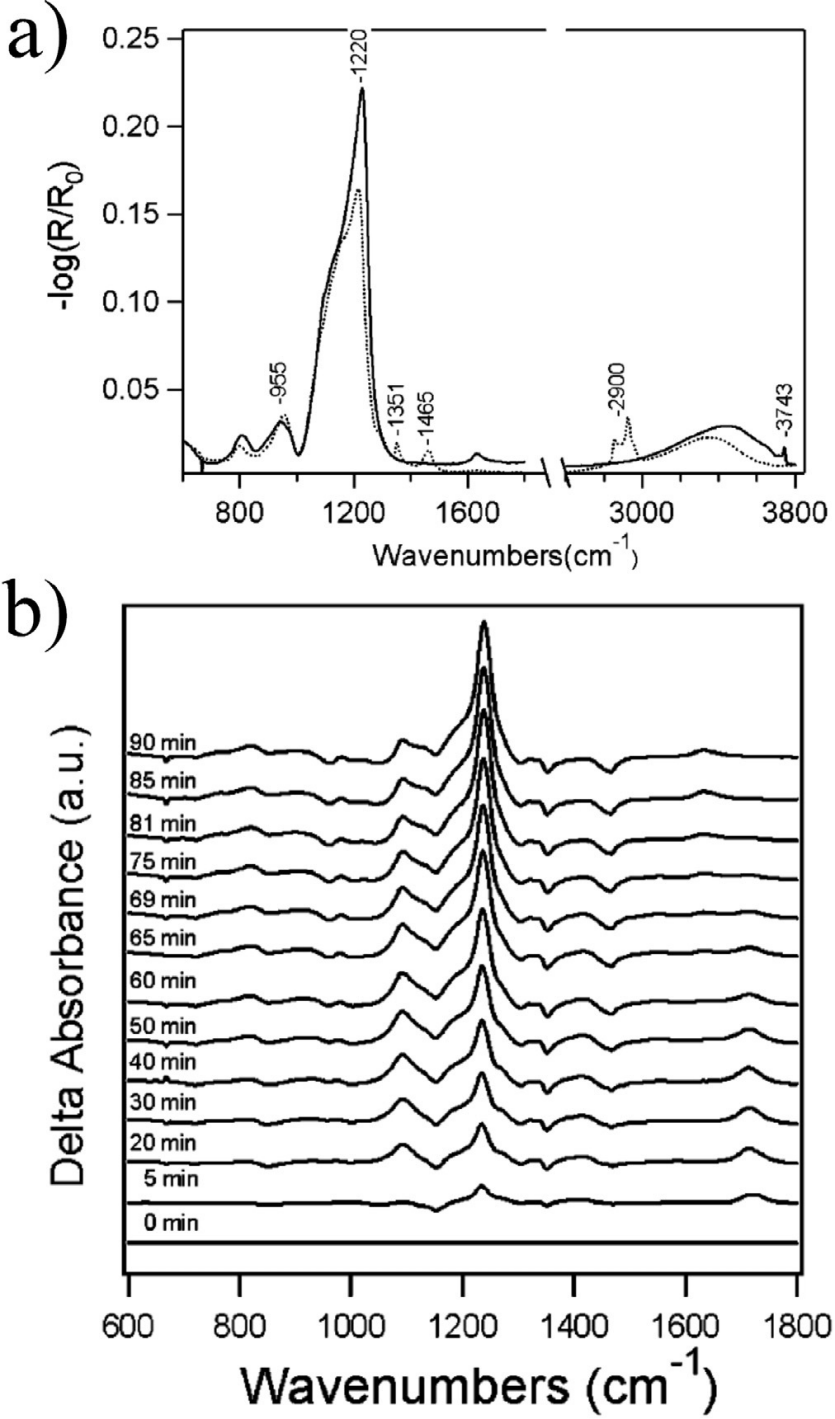
a) FTIR spectra of as‐deposited (Brij56 templated nanocomposite) SiO_2_ thin films before (dotted line) and after (solid line) exposure to DUV irradiation for 180 min. b) Evolution of the FTIR spectrum of an as‐deposited (Brij56 templated nanocomposite) SiO_2_ thin film as a function of exposure time to DUV irradiation. Reprinted with permission from ref. 41. Copyright 2005 American Chemical Society.

The application of UV light to improve the condensation among the metal precursors and the densification of the resulting metal‐oxygen network probably had its origins in the early studies of mesoporous thin film materials based on SiO_2_. Such materials are typically formed using a self‐assembled organic phase that templates the formation of inorganic silica by a templated sol‐gel synthesis process. This method requires the selective removal of the organic phase (surfactant) from the mesostructured thin film, which is usually accomplished by calcination at relatively high temperature (≤450 °C). To avoid the drawbacks associated with this step, such as the collapse of the mesostructured framework or the damage of a temperature‐sensitive metal substrate, a nominally room‐temperature photochemical method was originally proposed.^40^ It consists of a UV/ozone treatment on the thin film that effectively removes the organic template phase besides strengthening the silicate skeleton through increased silica condensation. This last effect is demonstrated in Figure 10, where grazing incidence FTIR spectra are shown for an as‐deposited templated nanocomposite (Brij56/TEOS) thin film subjected to UV/ozone treatment with a low‐pressure mercury lamp (*λ*=184–257 nm).^41^ The increase in the intensity of the Si‐O‐Si mode upon irradiation (Figure 10 a) would be related to the formation of more metal‐oxygen bonds within the inorganic network by promoting the condensation among silanol groups. Note how the intensity of this mode gradually increases with the time of exposure (Figure 10 b), thus confirming the silica condensation induced by this process at room temperature.

**Figure 11:**
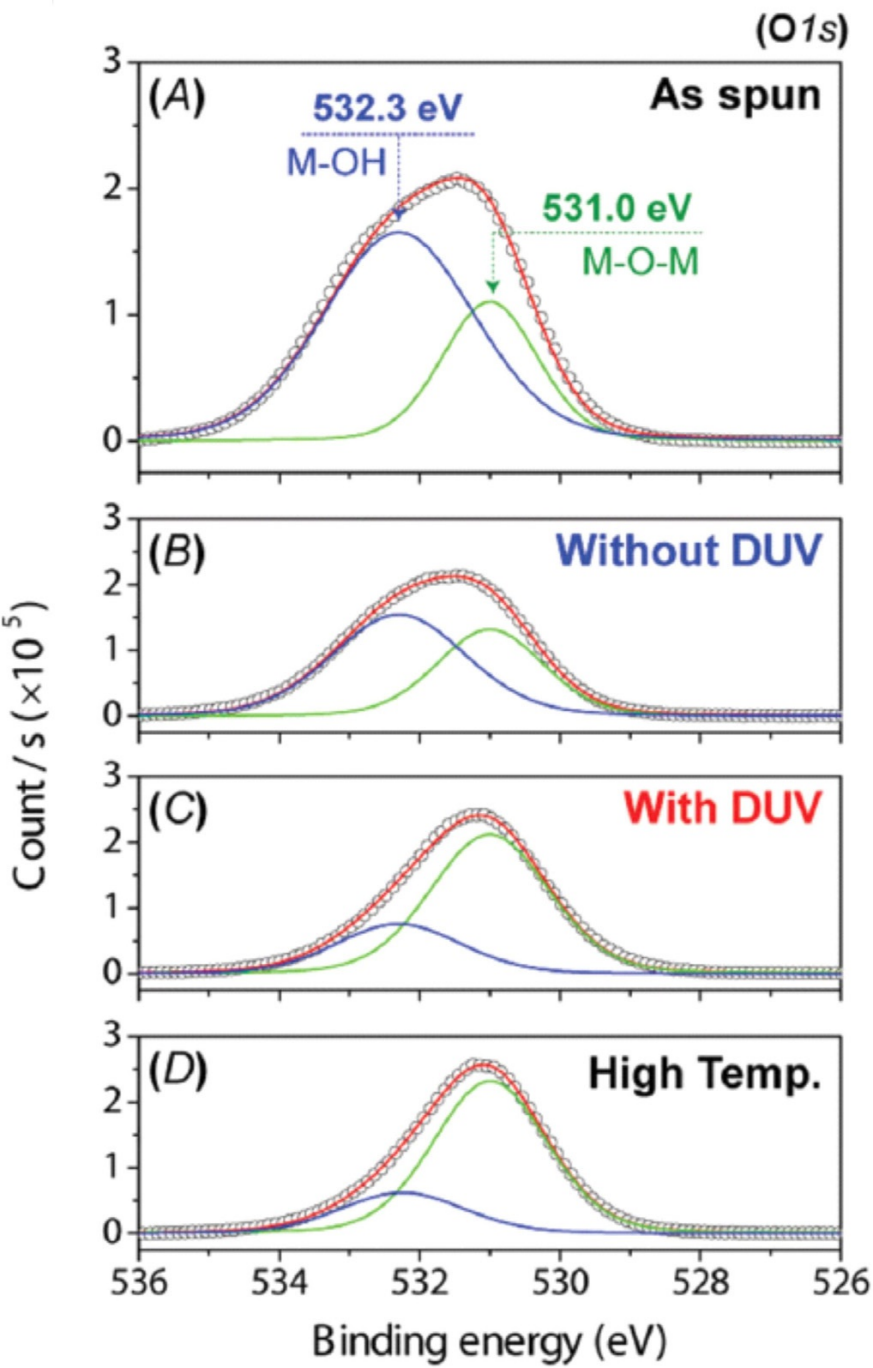
Deconvoluted O 1s XPS spectra of AlO*x* thin films prepared under different conditions: A) the as‐spun film, a film treated at 100 °C for 5 min B) without and C) with DUV irradiation, and D) a film thermally annealed at 350 °C for 60 min. Reprinted with permission from ref. 24. Copyright 2015 Wiley‐VCH.

Whereas the initial studies on mesoporous silica thin films shown before were mainly focused on preserving the structural morphology and surface characteristics of the material, the significant enhancement of polycondensation upon UV irradiation drew the attention of the semiconductor community a few years ago. As stated in the Introduction section, the performance of amorphous metal oxide semiconductors is strongly dependent on the formation of a defect‐free, highly densified metal‐oxygen network in the thin film.^10^ Irradiation with UV light would not only promote the condensation among metal precursors, but it would also enable the use of flexible polymeric substrates due to the low temperature associated with the film processing (near room‐temperature). High‐resolution X‐ray photoelectron spectroscopy (XPS) is usually undertaken to study the degree of polycondensation in metal oxide thin films together with their chemical composition. Figure 11 shows the Gaussian curve fits of the O 1s signal measured by XPS in solution‐processed Al_2_O_3_ thin films treated under different conditions.^24^ In all cases the O 1s signal is very broad, which suggests multiple oxygen environments in the system resulting in different chemical shifts. Thus, the oxygen peak can be fitted to a superposition of two Gaussian components with positions centered around 531.0 and 532.3 eV that reflect two different oxygen environments ascribed to M‐O‐M and M−OH species, respectively. The results clearly show that the film dried at 100 °C for 5 min without UV irradiation (without DUV) contains an appreciable concentration of M−OH species very similar to that observed in the as‐deposited film (as spun). In contrast, the film counterpart subjected to UV irradiation (with DUV) denotes a much larger contribution from the M‐O‐M signal, showing an XPS profile practically identical to a thermally annealed film at 350 °C (high temperature). From this analysis it can be easily deduced that the condensation among the metal precursors in the as‐deposited layer is significantly enhanced upon DUV irradiation.

**Figure 12:**
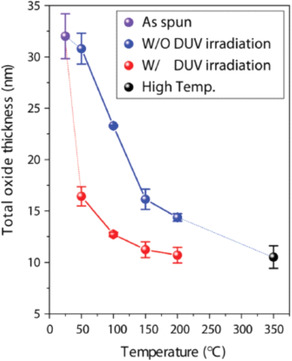
Effect of DUV irradiation in the thicknesses of AlO*x* thin films maintained at 150 °C. Reprinted with permission from ref. 24. Copyright 2015 Wiley‐VCH.

Besides promoting the condensation among the metal precursors, further irradiation of the metal‐oxygen network can also improve the densification of the metal oxide thin film. This effect occurs in a process parallel to that of polycondensation, with both process developing gradually and concomitantly with increasing the exposure to UV light. Figure 12 shows the thickness measured in several Al_2_O_3_ thin films annealed at different temperatures with and without UV irradiation.^24^ Thus, DUV exposure reduced the final thickness of oxide films annealed at 150 °C from 16 nm to 11 nm (non‐irradiated). This result is confirmed by many other works,^42^, ^43^, ^44^ suggesting that irradiation with UV light induces the tight packing of the initially coarse M‐O‐M network leading to dense films with comparatively low thicknesses. As it was described in the previous section, exposure to UV light enhances the removal of organic species from the system in a low‐temperature regime that results in the more uniform drying of the as‐deposited film with respect to thermal treatments at higher temperatures (e.g., pyrolysis). Here, the contraction of the gel network after solvent evaporation (due to capillary tension) critically determines the final pore volume in the thin film. Densification of the metal‐oxygen network can be therefore promoted by further condensation reactions among the chemical precursors that are brought now into closer contact after a controlled drying process induced by UV light. In addition to this structural effect, photogenerated species such as atomic oxygen can also react with suboxides and oxygen vacancies (V_O_^..^) present in the thin film thus improving the metal oxide stoichiometry and decreasing the density of crystal defects by compensation.^45^

### 3.4 Photoinduced nucleation and growth of crystalline metal oxides

Whereas the effects of UV irradiation on the low‐temperature processing of metal oxide thin films are primarily focused on the stages of the PCSD process described in the previous sections (decomposition of the metal precursors and condensation‐densification of the metal‐oxygen network), a small number of recent studies have demonstrated the validity of this approach to crystallize dissimilar inorganic materials such as zeolites or metal oxide nanostructures. In spite of their different composition or morphology, metal oxide thin films could be affected by the same mechanisms for promoting the nucleation and growth events once the following approaches were optimized and extrapolated to the corresponding metal oxide layers.

**Figure 13:**
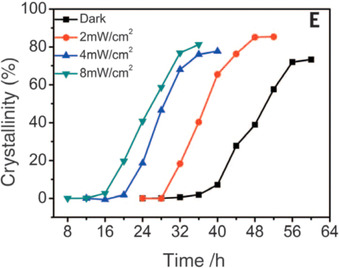
Crystallization curves of a zeolite without and with UV irradiation under different irradiance conditions. Reprinted with permission from ref. 46. Copyright 2016 ScienceMag.

In 2016, it was reported that hydroxyl free radicals (OH^.^) generated by UV irradiation can accelerate the crystallization of zeolites (microporous crystalline aluminosilicates) under hydrothermal conditions.^46^ Such radicals are originated upon irradiation of water in the media, and these species would be responsible for accelerating the kinetics of the nucleation stage. The crystallization curves of a zeolite compound (SiO_2_: 0.46 Al_2_O_3_: 4.4 Na_2_O: 60 H_2_O) in the absence of light and under UV light with different irradiances is shown in Figure 13. Whereas 60 hours were required to obtain a highly crystalline zeolite under dark conditions, this time was significantly reduced to 32, 20, and 16 hours for irradiances of 2.0, 4.0, and 8.0 mW cm^−2^, respectively. Considering a mechanism for zeolite crystallization based in two steps (depolymerization of the gel by breaking Si‐O‐Si bonds and subsequent formation of new Si‐O‐Si bonds), theoretical calculations yield lower activation energies for both processes when OH^.^ radicals (instead of only OH^‐^hydroxyl groups) are present in the media. Particularly, an enhanced positive effect of OH^.^ against OH^−^ was observed in the dissociation of the Si‐O‐Si bonds (4 versus 29 kcal mol^−1^) and formation of new Si‐O‐Si bonds (8 versus 17 kcal mol^−1^)when using the mono‐deprotonated [Si(OH)_2_‐O‐Si(OH)_3_]Na model of the gel. Although not explored yet, the proposed mechanism for zeolite crystallization could be potentially transferred to metal oxide thin films prepared under similar conditions (e.g., hydrothermal synthesis) after a proper implementation.

**Figure 14:**
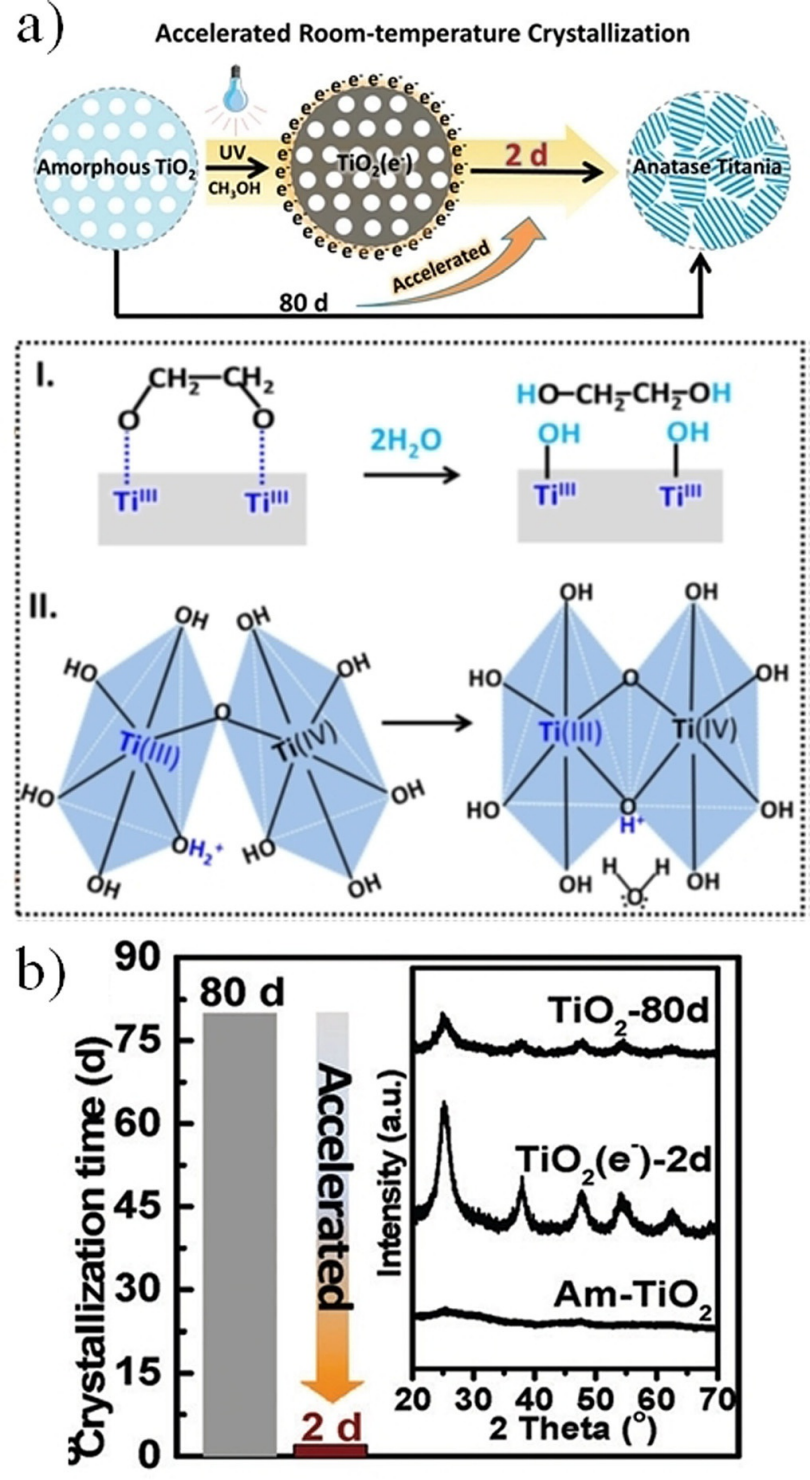
a) Schematic representation of the accelerated room‐temperature crystallization from amorphous to anatase TiO_2_. b) Crystallization time of TiO_2_ after 80 days (grey) and after storing electrons for 2 days (red). Inset shows the corresponding XRD patterns of the respective TiO_2_‐80d and TiO_2_(e^−^)‐2d samples. Reprinted with permission from ref. 26. Copyright 2017 The Royal Society of Chemistry.

The photoinduced synthesis of nanostructured metal oxides has been reported in the last few years using energy‐efficient processes at room temperature. For example, crystallization of TiO_2_ anatase from amorphousTiO_2_ can be significantly accelerated by storing in the material photogenerated electrons from UV irradiation.^26^ A suspension of amorphous TiO_2_ powder in aqueous methanol, which works as a sacrificial agent to capture the photogenerated holes, was subjected to UV light for 30 min generating abundant electrons on the surface of the titania powders. After maintaining the photoactivated suspension for several days, a solid product corresponding to crystalline TiO_2_ anatase was collected after drying and washing of the corresponding dispersion. Figure 14 shows the X‐ray diffraction (XRD) patterns of different TiO_2_ samples obtained by this method, together with a scheme depicting the process. Note that amorphous TiO_2_ requires 80 days to convert into crystalline TiO_2_ anatase at room temperature (Figure 14 b), whereas this time is abruptly reduced down to 2 days after storing photogenerated electrons in the precursor (photoactivated suspension). The underlying mechanism is explained in the following terms (Figure 14 a): (I) first, the storage of electrons reduces some Ti^4+^ to Ti^3+^ weakening the Ti−O bond between titania and the residual solvent (ethylene glycol), which is easily removed from the TiO_6_ octahedra leaving hydroxyl groups exposed on the titania surface; (II) then, a proton can be attached to the surface oxygen after the combination with an electron stored in the neighboring Ti^3+^. These protonated surfaces would easily interact with the hydroxyl groups of other TiO_6_ octahedra releasing a H_2_O molecule and forming a bridging oxygen bond (Ti‐O‐Ti) between the two neighboring octahedra.

**Figure 15:**
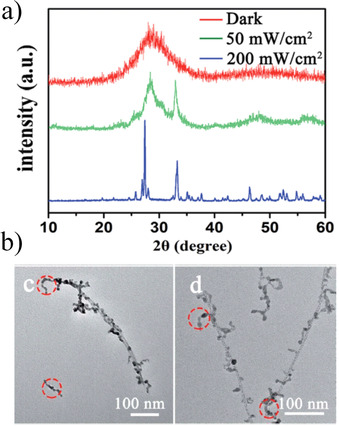
a) XRD patterns of amorphous bismuth hydroxide nanoparticles under dark conditions for 1 hour and their crystallization into α‐Bi_2_O_3_ nanotubes under UV conditions for 1 hour with different light irradiances. b) TEM images showing the intermediate states between α‐Bi_2_O_3_ nanoparticles and nanotubes. Reprinted with permission from ref. 25. Copyright 2019 The Royal Society of Chemistry.

Very recently (2019), irradiation with UV light has also been demonstrated to promote the crystallization of α‐Bi_2_O_3_ nanotubes from amorphous bismuth hydroxide at room temperature.^25^ Ultraviolet treatments were conducted on an amorphous bismuth hydroxide precursor dispersed in aqueous media with irradiation times of 1 h (Xe lamp, 420 nm cutoff filter). Figure 15 shows how the XRD pattern of the product remains amorphous under dark conditions (Figure 15 a), whereas under UV conditions using light irradiances of 50 and 200 mW yields α‐Bi_2_O_3_ nanotubes and bulk α‐Bi_2_O_3_ according to their respective TEM images (Figure 15 b). In this work, UV irradiation can not only induce the crystallization of bismuth oxide at room temperature, but it also controls the morphology of α‐Bi_2_O_3_ by means of the intensity of the UV light employed. Similar to the previous example on the crystallization of TiO_2_ anatase, the electronic excitation of the Bi−O bond present in the amorphous bismuth hydroxide (whereby a photoinduced charge transfer of electrons from oxygen to bismuth is produced) would facilitate the formation of Bi‐O‐Bi bridges after the elimination of a water molecule. Thus, UV irradiation promotes the dehydration and condensation of amorphous bismuth hydroxide to crystalline α‐Bi_2_O_3_.

## 4 Summary and Outlook

In this review, we have shown the chemical effects of light irradiation (mainly, ultraviolet) at the different stages of the chemical solution deposition of metal oxide thin films. Illumination with solar or UV lamps can be carried out in both the precursor solution and the solution deposited layer. This makes possible to induce photochemical reactions in the chemical system (solution, thin film and atmosphere)leading to 1) the synthesis of low‐temperature liquid precursors, 2) an enhanced decomposition of metal precursors and elimination of organic residuals, 3) a high degree of condensation and densification of the metal‐oxygen network, and 4) a prompt nucleation and growth of the crystalline oxide phase. The formation of a close‐packed metal‐oxygen network in the amorphous film and its subsequent crystallization would be therefore advanced to a lower temperature range(<350 °C), enabling its direct integration as an active layer into high‐performance flexible electronic systems besides opening the door to a plethora of applications for metal oxide thin films today hindered by their relatively high crystallization temperatures. In addition to this technological advantage, the reduction of the processing temperature would also provide environmental and economic benefits derived from the reduction of the energy consumption of the whole manufacturing process.

The types of chemical reactions induced by light in organic compounds are well described in the literature,^2^ in contrast to those that can be produced in inorganic materials. This is because the range of photochemical reactions is scarcer in the latter systems. Actually, irradiation with UV light began to be used in the fabrication of solution‐derived metal oxide thin films due to the large amount of organic species present in the as‐deposited layers. Since light penetration in solids is limited to a few hundred nanometers, reactions induced by light are always confined to the surface of the material. This phenomenology therefore entails a great challenge as well as an opportunity for using photochemistry in the low‐temperature processing of solution derived metal oxide thin films. Thus, the absorption of light in the as‐deposited film results in photochemical reactions such as photolysis and photoinduced charge transfer, and the generation of reactive species (e.g., active radicals) that overall lead to the cleavage of chemical bonds, condensation and densification of the system and even the formation of the first crystal nuclei. The extent of the crystallization throughout the film needs however the rearrangement of the metal and oxygen atoms. This involves diffusion processes that are typically slow in solids and require very long times to attain full crystallization in the film using only photochemistry. Consequently, a small amount of energy has to be supplied to the system that is usually provided by a gentle heating at temperatures well below the values conventionally required in the absence of any photochemical assisted method. The prospect of a sustainable fabrication process for metal oxide thin films with practically null heating demands may look distant today, but we believe that the shorter road to it goes unavoidably through photochemistry.

To conclude, with this review we aim to convey the great potential of photochemistry for the low‐temperature processing of metal oxide thin films by solution methods. Photochemical methods open novel pathways to synthesize thermodynamically disfavored chemical species and to overcome energy barriers otherwise not accessible with low thermal budgets. This can push the chemical system far from its equilibrium making possible the fabrication of metal oxide thin film at temperatures well below the values typically applied by conventional thermal treatments.

## Conflict of interest

The authors declare no conflict of interest.

## Biographical Information

*Íñigo Bretos is currently a Ramón y Cajal Fellow at the Materials Science Institute of Madrid (ICMM‐CSIC). He received his PhD in Chemistry from Univ. Autónoma de Madrid in 2006. He then joined RWTH Aachen University (Germany) as a postdoc. His research interests are focused on the processing and integration of functional oxide layers (ferroelectrics, piezoelectrics, dielectrics, multiferroics or superconductors) in advanced electronics and emerging technologies for applications ranging from memories, sensing and transduction to photovoltaics and flexible systems. Over the years, his research career has evolved from the area of microelectronics to the field of flexible electronics*.

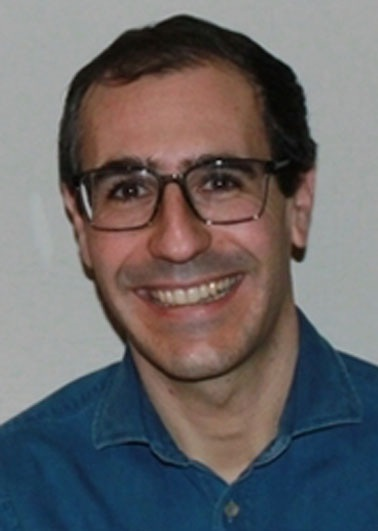

## Biographical Information

*Ricardo Jiménez, senior scientist at ICMM‐CSIC, obtained his PhD in Chemistry from Univ. Autónoma de Madrid in 1994. He made a postdoctoral stay at the “Laboratoire de electrochimie et physico‐chimie of solids and interfaces” (1995–1997) in Grenoble (France). His foundation in solid‐state electrochemistry, together with his experience in ferroelectric materials characterization, contributes to his expertise in the functional characterization of electro‐ceramics (both bulk and thin films). His research covers the development of novel techniques for functional materials preparation and characterization including materials for electrochemical energy storage and generation*.

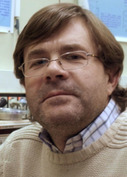

## Biographical Information

*Jesús Ricote received his PhD degree in Physics from Univ. Complutense de Madrid in 1994. After a postdoctoral stay at the Centre d'Elaboration et des Études Estructurales de Materiaux, CNRS (Toulouse, France) in1995, he joined the Nanotechnology Group of Cranfield University (UK) as a research officer (1996–1998). Later he obtained a postdoctoral fellowship (1998–99) at the Univ. du Maine‐Le Mans (France). In 1999 he joined the Materials Science Institute of Madrid (CSIC) and became Tenured Scientist in 2007. His research interests focus on stablishing the role that the microstructural elements play at different scales in the behavior of polycrystalline ferroelectric materials*.

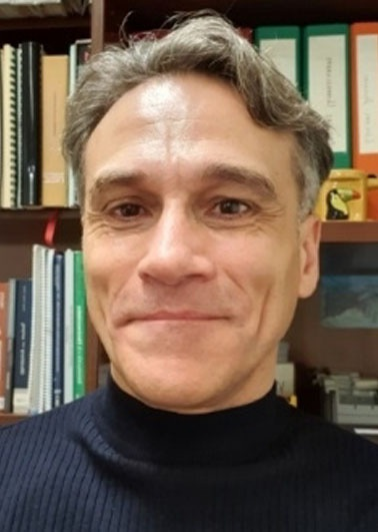

## Biographical Information

*M. Lourdes Calzada is Full Professor at the Institute of Materials Science of Madrid (ICMM‐CSIC). Her projects are focused on the “Development of low‐temperature sol‐gel synthesis strategies to attain metal oxide materials” and on the “Integration of functional thin films with semiconductor and flexible substrates (Si‐technology and Flexible Electronics)”. Her group is pioneer in the “Low‐temperature solution processing of ferroelectric and multiferroic complex oxides for flexible electronic devices”. She has supervised six Ph.D Theses, published 190 papers and presented 15 invited talks in International Conferences, 10 of which in the field of the low‐temperature processing of films*.